# Improving the generalizability of white blood cell classification with few-shot domain adaptation

**DOI:** 10.1016/j.jpi.2024.100405

**Published:** 2024-11-07

**Authors:** Manon Chossegros, François Delhommeau, Daniel Stockholm, Xavier Tannier

**Affiliations:** a Sorbonne Université, Inserm, Universite Sorbonne Paris-Nord, Laboratoire d'Informatique Médicale et d'Ingénierie des Connaissances en e-Santé, LIMICS, 15 Rue de l'École de Médecine, 75006 Paris, France; b Sorbonne Université, Inserm, Centre de Recherche Saint-Antoine, CRSA, Paris 27 rue de Chaligny, 75012 Paris, France; c PSL Research University, EPHE, Paris 4-14 Rue Ferrus, 75014 Paris, France

**Keywords:** Deep learning, Classification, White blood cell, Few shot learning, Domain adaptation

## Abstract

The morphological classification of nucleated blood cells is fundamental for the diagnosis of hematological diseases. Many Deep Learning algorithms have been implemented to automatize this classification task, but most of the time they fail to classify images coming from different sources. This is known as “domain shift”. Whereas some research has been conducted in this area, domain adaptation techniques are often computationally expensive and can introduce significant modifications to initial cell images. In this article, we propose an easy-to-implement workflow where we trained a model to classify images from two datasets, and tested it on images coming from eight other datasets. An EfficientNet model was trained on a source dataset comprising images from two different datasets. It was afterwards fine-tuned on each of the eight target datasets by using 100 or less-annotated images from these datasets. Images from both the source and the target dataset underwent a color transform to put them into a standardized color style. The importance of color transform and fine-tuning was evaluated through an ablation study and visually assessed with scatter plots, and an extensive error analysis was carried out. The model achieved an accuracy higher than 80% for every dataset and exceeded 90% for more than half of the datasets. The presented workflow yielded promising results in terms of generalizability, significantly improving performance on target datasets, whereas keeping low computational cost and maintaining consistent color transformations. Source code is available at: https://github.com/mc2295/WBC_Generalization

## Highlights

• Our study introduces a workflow for classifying blood cells from different domains.
• The model adapts to eight datasets with minimal annotations (less than 100 images each).
• We implement color standardization in lαβ space for consistent visual style.
• Results show good generalization, with accuracy exceeding 90% for many datasets.

## Graphical abstract

**Figure:**
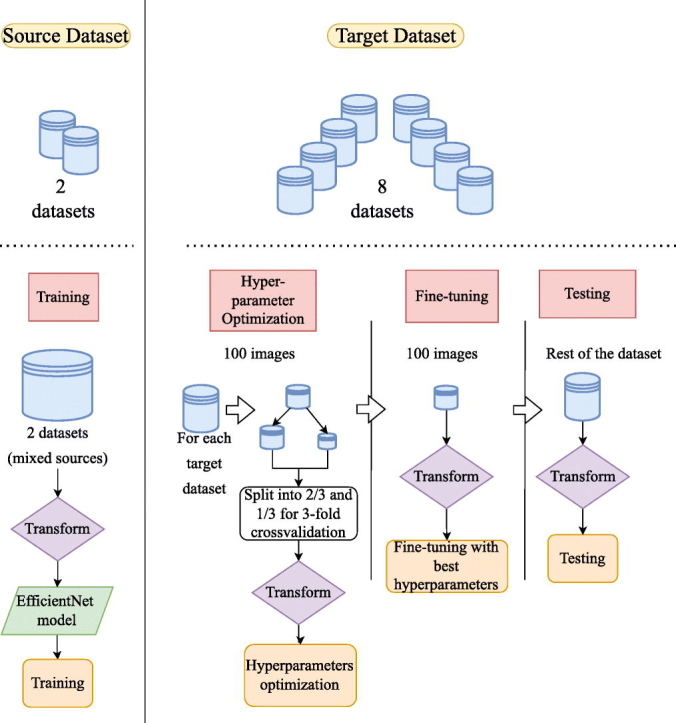

## Introduction

### Context

The study of blood smears is a routine laboratory test that follows automated complete blood cell count and leucocyte differential count. It provides essential information about the patient health condition. Nucleated blood cells are divided into different categories including white blood cells and occasionally nucleated red blood cells (or erythroblasts). Differential count is essential to reveal disorders that affect specific types of cells. Therefore, the classification of white blood cells and nucleated red blood cells is a key stage for the diagnosis of various pathological conditions, including malignancies such as leukemia and lymphomas. Usually, this analysis is performed by experts who visually assess microscopic images, which is error-prone and time-consuming.^1^ Deep learning solutions have gained significant importance in recent years, as they can automate processes and alleviate the increasing pressure on hospitals.^2^^,^^3^

A well-known problem with such an approach, however, is that it lacks generalizability capacity, and whereas a computer vision algorithm can give good results when trained on a given *source dataset*, it will most probably fail to obtain similar performance when applied on images from a different *target dataset.*^4^ Having images from different origins for training and testing can cause serious limitations. This phenomenon is called domain shift and is caused by variations in data distribution originating from different sources.^5^ This situation is very common; the visual aspect of images from various healthcare centers can vary greatly due to variations in lighting conditions, camera characteristics, backgrounds etc. and thus there is a need for algorithms that operate on data from any source. Domain adaptation is defined as the process of making an algorithm able to perform well on a new domain.

There has been a growing appeal for few-shot learning strategies applied to clinical data because the number of health-related labeled samples is most often scarce.^6^, ^7^, ^8^ In few-shot learning, the model is trained by using only a few annotated images. It can use previous knowledge learnt from another task, this process being defined as transfer learning. So far, only a few articles have tackled the matter of generalizability in the classification of white blood cells, and to our knowledge, no study has yielded an interpretable and simple-to-implement workflow for the classification of cell images coming from an unknown dataset.

In this article, we enhance the generalizability capacity of a classifier model on a new dataset, using a few labeled images. We propose a general workflow to classify images at low computational cost. We trained a neural network to classify white blood cell images from two *source* public datasets. Then, we applied the model to eight *target* datasets, by fine-tuning the model with no more than 100 labeled images from these datasets. For each dataset, a visual transformation was applied to standardize the color appearance to a common style. We also conducted extensive experiments to evaluate and understand the role of each step of our process. We visually assessed the benefits of our approach, and we carried out an error analysis. This article studies a large number of data sources, some of the aforementioned datasets having never been studied for generalizability performances before. This shows that a clinical center willing to classify a local dataset of images can obtain good performance with little annotation effort.

### Related work

Until recently, the early diagnosis of hematological disorders was mainly based on clinician visual assessment of blood smears. The digitization of images, however, has paved the way for automated processes.^1^

At first, the automation of image treatment solely relied on machine learning principles such as SVM or random forest.^9^^,^^10^ This approach needed consequent pre-processing of the images, to extract relevant features before training models. In particular, the segmentation of the cell or the nucleus was often necessary.^11^^,^^12^

More recently, deep learning solutions have gained increasing popularity due to their ability to automatically extract relevant features without the need for manual pre-processing.^2^^,^^3^^,^^13^ Many studies have explored the classification of different cell types from peripheral blood or from bone marrow.^4^^,^^8^^,^^14^, ^15^, ^16^, ^17^ Classification was also employed for the differentiation between healthy and pathological cells.^6^^,^^18^^,^^19^

Most of the previously mentioned studies utilized uniform datasets, with both the training and testing phases employing images from the identical dataset. Nevertheless, a few articles addressed the issue of model generalizability, with most of them employing solely two or three different datasets. Among them, some researchers have dealt with generalizability by producing new images. In Refs,^20^, ^21^, ^22^, ^23^ generative adversarial network (GAN) models were designed to generate different-looking images and to augment the size of the dataset. Furthermore, data augmentation methods were employed, but mainly with random augmentation techniques, such as color jittering or random crop.^24^ In Baydilli et al.,^20^ and Claro et al.,^24^ 10 datasets and 18 datasets were employed, respectively, in order to enhance the diversity of images. To the best of our knowledge, these are the only articles employing more than five datasets for the classification of white blood cell types.

At a feature level, other studies have tried to extract domain-independent features from images. In Refs,^25^, ^26^, ^27^ machine learning and deep learning techniques were combined to extract more robust features. This needed, however, further preprocessing treatment. Other articles trained models to specifically extract generalizable features in parallel with classification; in this case, the adversarial loss was used to make feature vectors indistinguishable.^28^^,^^29^ Nevertheless, this approach was still specific to the domains under study.

Combining feature and image focus, another approach was developed in Pandey et al.^30^; a variational autoencoder was trained to reconstruct an image from a wide latent space and classify it. When a new dataset was considered, the closest clone was chosen in the latent space thanks to structural similarity index, and the reconstructed image of this clone was classified.

These approaches presented several limitations. First, in order to generalize well, some of these models needed images from the test dataset for training. Second, the computational cost was high. Finally, whether it was with GAN or image transforms, the creation of new images did not ensure they were realistic looking.

## Material and methods

### Overview

**Fig. 1:**
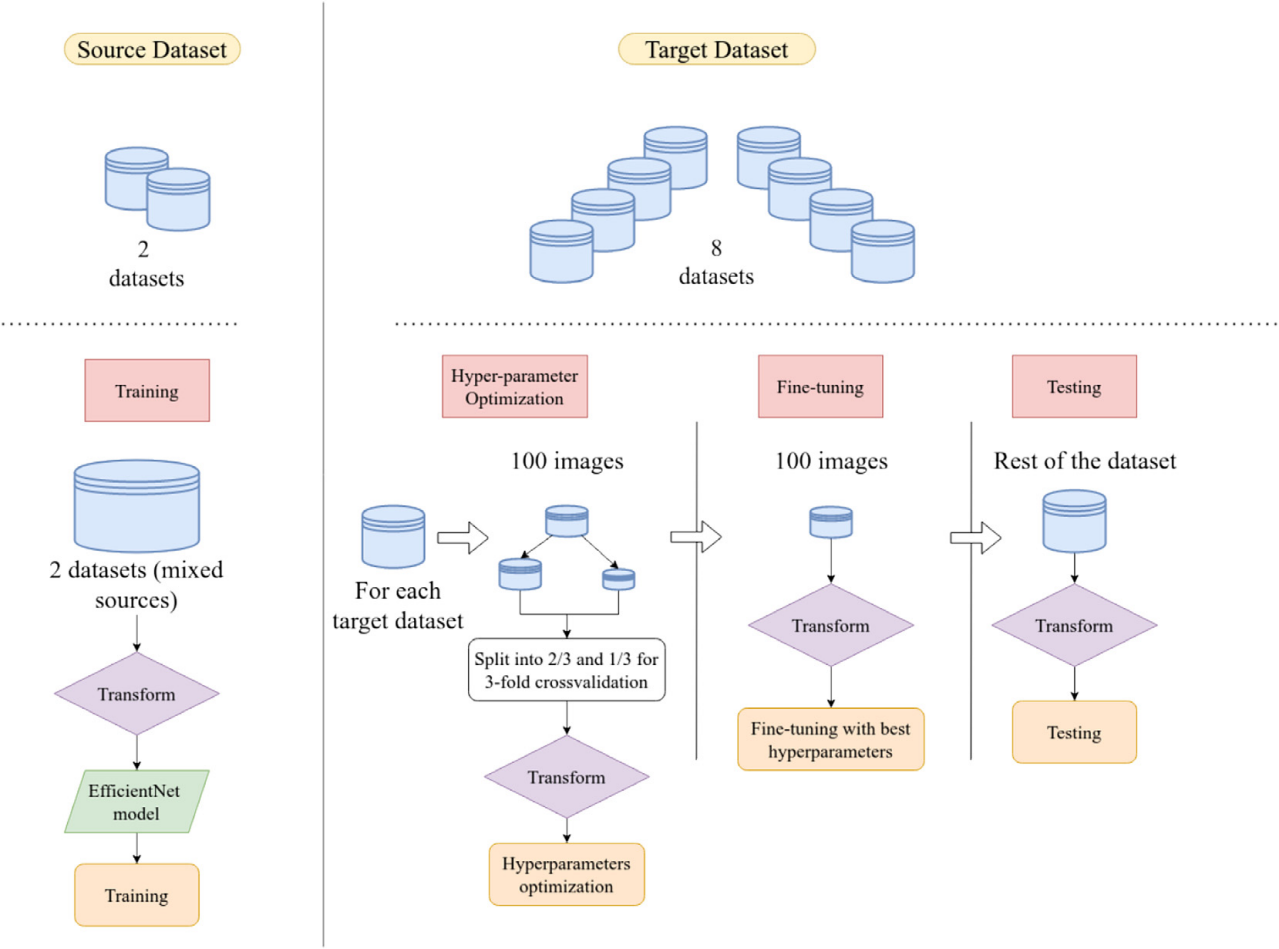
Overview of the general workflow. Firstly, the model was trained on two source datasets. Secondly, for each target dataset, it was fine-tuned on 100 or less images and tested on the remaining images. All images underwent visual transform.

The overall workflow of our study is illustrated in Fig. 1, and consists in using different datasets for training and test, in order to evaluate the out-of-domain performances of our model.

First, a model employing the EfficientNet architecture was trained on two source datasets: it constituted the *source* dataset. Images were collected and made public by Barcelona and Munich clinical centers.^31^^,^^32^ Afterwards, we fine-tuned the model on the eight other datasets, called the *target datasets*. For each *target* dataset, fine-tuning hyperparameters such as learning rate or batch size were optimized by dividing the fine-tuning dataset into train and valid subsets, and employing cross-validation. We report the numbers of images selected for fine-tuning in Table 3. The rest of the *target datasets* were used for testing purpose. A transform was applied both to the source and the target images to ensure they all followed the same visual “style”.

### Description of the datasets

**Fig. 2:**
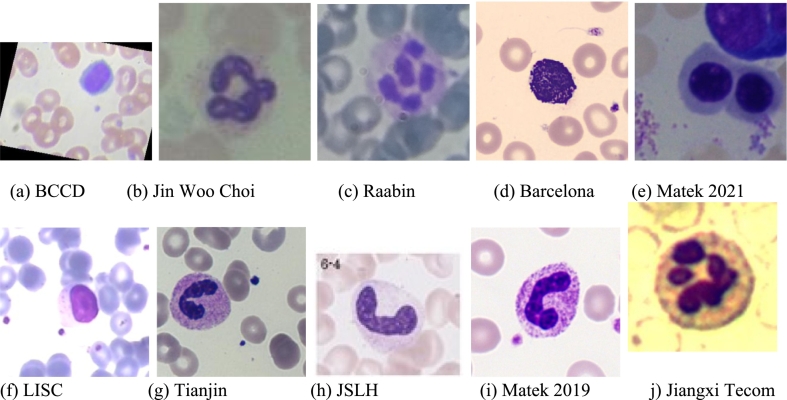
Images from every dataset. Images come from 10 different dataset, both of bone marrow and of peripheral blood cells.

After a review of existing datasets in literature, we collected images from 12 studies. From these datasets, we discarded Cella Vision Blog which was very small and contained several annotation mistakes, as well as Ruinjing dataset which was only composed of lymphocytes, lymphoma, and blasts. Finally, we used 10 datasets, 2 of them being used for training, and the rest for fine-tuning and testing. The description of the final datasets is given in Table 1, and sample images from each dataset are shown in Fig. 2. The visual aspects of the images varied among the different datasets due to resolution, capture method, and magnification power. Details about the acquisition conditions for each dataset are provided in Appendix A. Both peripheral blood smears and bone marrow smears are represented in datasets and they were treated the same by the algorithm. For this study, only six WBC classes were considered, namely eosinophils, basophils, neutrophils, monocytes, erythroblasts, and lymphocytes. These classes were chosen as to maximize the number of classes shared among datasets, regardless of where cells were from. In some cases, classes had higher granularity; for example, neutrophils could be described as band neutrophil or segmented neutrophils. We classified these images into the main category they belonged to; in this case, they would both be labeled as neutrophils.

**Table 1:** Description of the datasets with the links to the datasets. Tianjin dataset is available upon request. Classes used for this study are bolded. Only basophils, eosinophils, erythroblasts, lymphocytes, monocytes, and neutrophils were kept for study. If these classes were described more precisely (e.g., band neutrophils and segmented neutrophils) only the main label (e.g., neutrophil) was given to the cell.

Name	Nb images	Classes
Barcelona^31^	17,092	**NEU**, **EOS**, **BAS**, **LYT**, **MON**, PMO, MYB, **EBO**, PLA, THO
Jiangxi Tecom^33^	300	**BAS**, **EOS**, **LYT**, **NEU**, **MON**
Jin Woo Choi^34^	2174	**NGB**, **NGS**, **ERB**, MMZ, MYO, MYB, **ERO**, **ERP**, PMO, PBL
BCCD^35^	3500	**EOS**, **LYT**, **MON**, **NEU**
LISC^36^	257	**BAS**, **EOS**, **LYT**, **NEU**, **MON**
Munich 2021^4^	171,373	**NGB**, **NGS**, **LYT**, **MON**, **EOS**, **BAS**, MMZ, MYB, PMO, BLA, PLM, KSC, OTH, ART, NIF, PEB, **EBO**, HAC, ABE, **LYI**, FGC
Munich 2019^32^	18,365	**BAS**, **EBO**, **EOS**, KSC, **LYT**, LYA, MMZ, MYO, **MON**, **NGB**, **NGS**, PMB
Raabin^37^	4514	**EOS**, **LYT**, **MON**, **NEU**
JSLH^38^	148	**NGB**, **ERB**,MMZ,MYO, **ERO**, **ERP**, PEB, PMO, **NGS**
Tianjin^39^	8564	**BAS**, **EOS**, **LYT**, **NEU**, **MON**

Table 2 details the number of available images. The names of cells categories are given by a three-letter abbreviation. The corresponding full names can be found in the Glossary.

**Table 2:** The distribution of cell classes for every dataset.

Dataset	Basophils	Eosinophils	Erythroblasts	Lymphocytes	Monocytes	Neutrophils
Munich 2021	441	5891	27,395	26,307	4040	39,392
Rabin	301	1066		3609	795	10,862
Munich 2019	79	424	78	3948	1789	8593
Lisc	53	39		52	48	50
Jslh			56			24
Jin woo choi			150			100
Jiangxi tecom	1	22		48	53	176
Bccd		3133		3108	3095	3171
Tianjin	302	1098		1863	1201	4100
Barcelona	1218	3117	1551	1214	1420	3329

### Data processing

Images from Tianjin and LISC datasets were not initially centered around the cell. Thus, these images were first cropped to have a 250 × 250 window around the cell. Masks were provided with LISC images, and bounding boxes with Tianjin images.

**Fig. 3:**
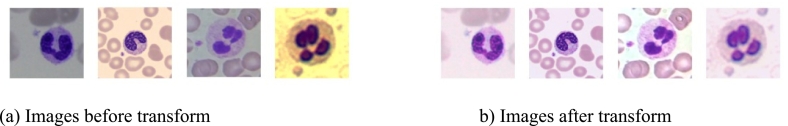
Color transform: all images were put in the same style with Lab-space transformation.

**Fig. 4:**
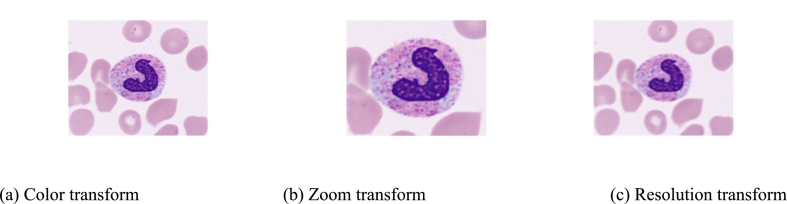
Successive transforms applied to training set.

We applied a color transform to standardize the color style of each image (Fig. 3). Color-based transformation was chosen, and more precisely conversion with *lαβ* space – also known as CIELAB color space –^40^ following the method described in Reinhard et al.^41^ In addition, to better represent the variety of zooms and of resolutions, images from Barcelona that were of better quality in the training set were augmented with degradation of image resolution and random zoom (Fig. 4). After these transformations, all the augmented images were resized to 224 × 224 size as input of the neural network.

### Training strategy

**Fig. 5:**
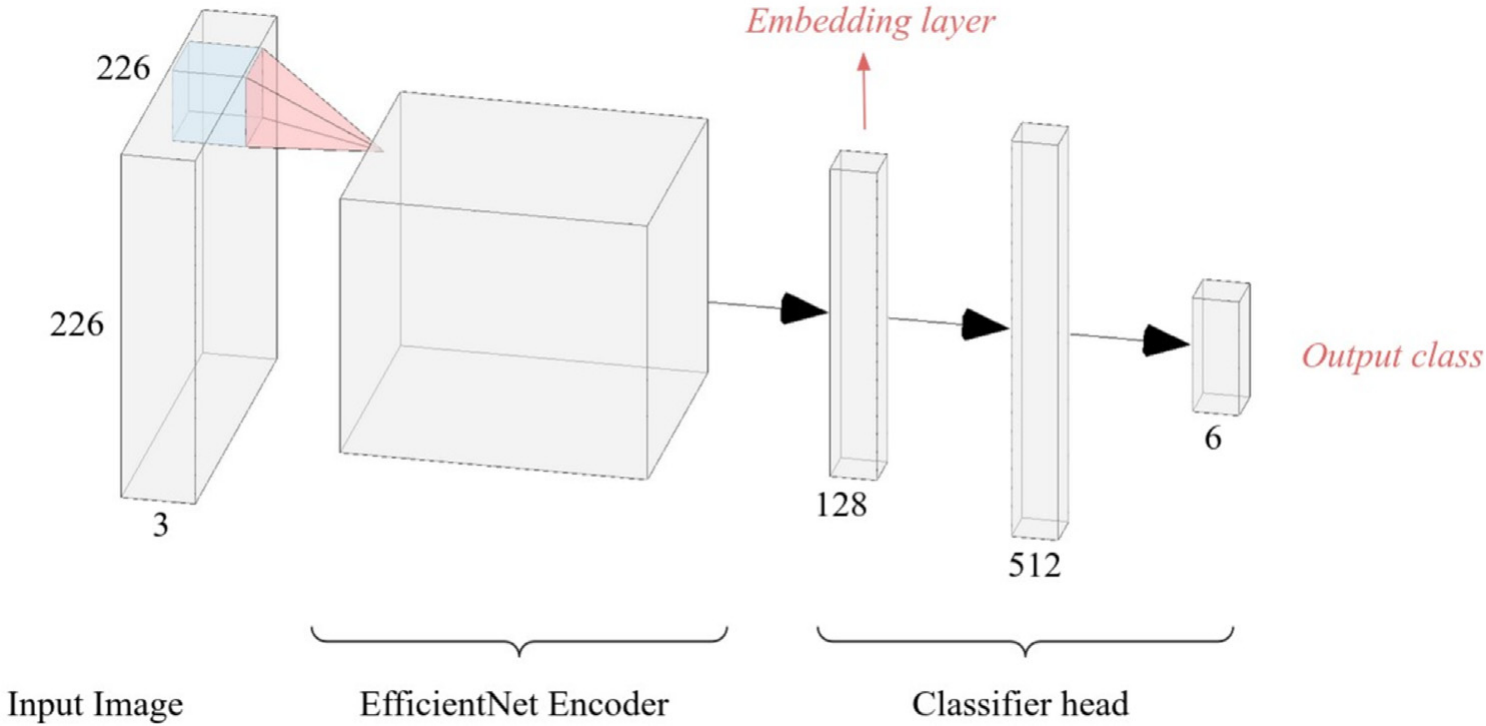
Model architecture. The EfficientNet Encoder extracts the features from the image. These features are represented in the 128-layer, which we will refer as the image embeddings. The three fully connected linear layers are called head of the model and perform classification.

A preliminary comparison was drawn between the following architectures: VGG16, ResNet101, EfficientNet-B0, ViT, and Inception V3, in order to determine the model that would exhibit the best performances on out-of-domain images. This evaluation was based on a small subset of images. EfficientNet architecture was selected for an overall stronger ability to generalize across diverse datasets. The model was divided into two parts: the encoder was designed to extract features from images, and was implemented according to EfficientNet-B0 architecture. At the end of the encoder, a 128-node linear layer was added. We referred to the output of this layer as the image embedding. Then, a classifier head was positioned at the top of the model, comprising two fully connected linear layers, with 512 and 6 nodes, corresponding to the number of classes. The classifier head also incorporated Batch Normalization and Dropout techniques, with the activation function ReLU applied (Fig. 5).

The training dataset was made of images from Barcelona and Munich 2019.^31^^,^^32^ The model was trained with batches made of images from these two datasets. The batch size was 16, the learning rate was 10^−4^ and the number of epochs was 15. LabelSmoothingCrossEntropy was chosen as a loss, and Adam optimizer was used. Computations were performed using Pytorch and Fastai library.

### Fine-tuning strategy

To reproduce the situation of a new clinical center coming with a few annotated images, 100 or less images were selected from every *target* dataset, with the same proportion of images of each class. Fine-tuning was performed by retraining the model using only these images, as if the clinical center had only this small subset as ground-truth. The number of images used was reported in Table 3. No images were needed to fine-tune the model on JSLH dataset because images were good enough to be classified without fine-tuning.

**Table 3:** Number of images used for fine-tuning. 100 images were taken from large datasets, 1/10th of the total number of images were taken for smaller datasets. No images were needed to fine-tune the model on JSLH, because images were already well classified without fine-tuning.

Fine-tuning set	Fine-tuning set size	Remaining images
Rabin	100	16,533
Munich 2021	100	103,366
Lisc	20	222
Jslh	0	72
Jin woo choi	20	230
Jiangxi tecom	25	275
Bccd	100	8511
Tianjin	100	2371

The choice of fine-tuning parameters, principally the learning rate, the batch size and the number of epochs, plays a critical role in the performance of the model. Given the limited size of the fine-tuning subsets and the important differences between images from different datasets, parameters had to be carefully set to prevent overfitting. Thus, the fine-tuning set was partitioned into training and validation subsets, and hyper parameter optimization was carried on using Optuna library. 3-fold cross-validation was utilized to mitigate the specificity of the results to the very small fine-tuning validation set.

## Results and discussion

### Classification of target datasets

The overall accuracy is defined as the number of rightly predicted images over the total number of images. Precision and recall are relative to a class.

$$\text{Accuracy}=\frac{\text{Rightly Labeled Images}}{\text{Total}\;\mathrm{Nb}\;\text{of Images}}$$

$$\text{Precision}=\frac{{\mathrm{TP}}_{\text{class}}}{{\mathrm{TP}}_{\text{class}}+{\mathrm{FP}}_{\text{class}}}$$

$$\text{Recall}=\frac{{\mathrm{TP}}_{\text{class}}}{{\mathrm{TP}}_{\text{class}}+{\mathrm{FN}}_{\text{class}}}$$

We first trained the model on *source datasets.* We split the two *source datasets* in 80/20 to evaluate the performances of the EfficientNet model after initial training*,* and obtained accuracy, macro precision and macro recall of 0.88, 0.76, 0.77 for Munich 2018 and 0.98, 0.98, 0.98 for Barcelona, respectively.

**Fig. 6:**
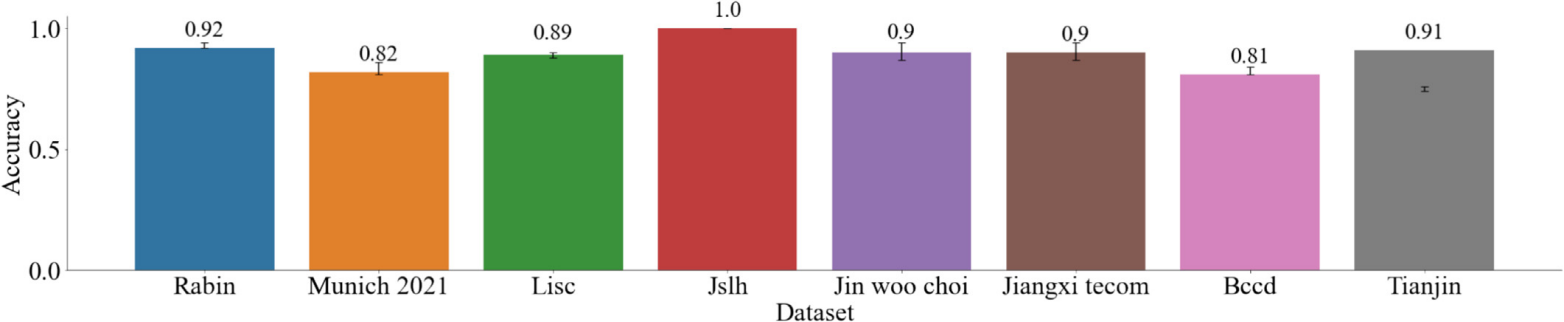
Accuracy per dataset, the error bars are the interval of confidence obtained with bootstrapping.

**Fig. 7:**
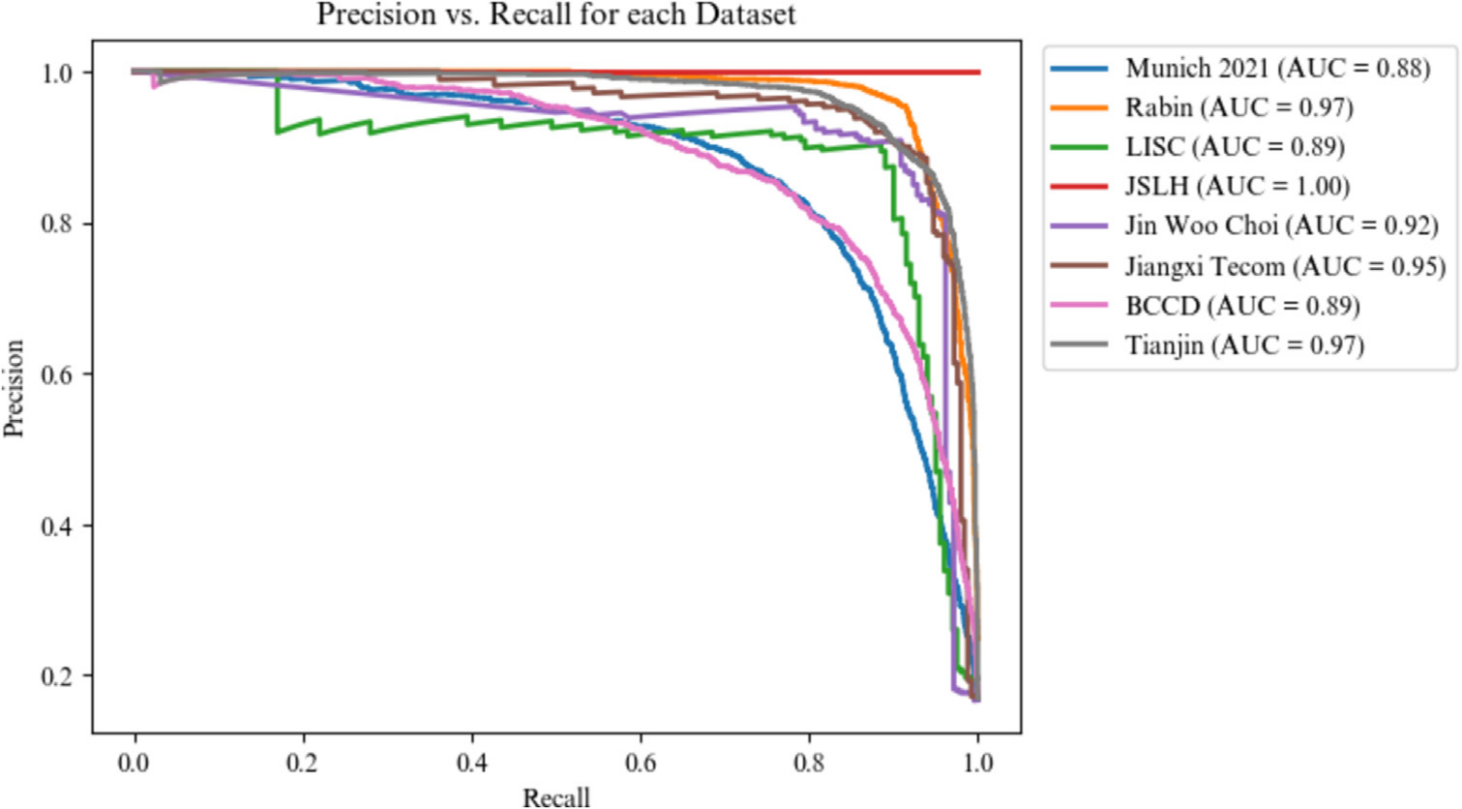
Precision vs Recall curve of the model predictions after fine-tuning on each dataset. Each curve averages the results over all classes.

Afterwards, the model was fine-tuned on each *target dataset*. The classification results of every fine-tuned model on its *target dataset* are reported in Fig. 6, and average Precision vs. Recall curves of each *target dataset* are plotted in Fig. 7. Detailed Precision vs. Recall curves can be found in Appendix B.

Bootstrapping techniques were employed on the model predictions to obtain confidence intervals of the results.^42^

The model accuracy is higher than 0.8 for each *target* dataset, showcasing its generalization capabilities despite the utilization of fewer than 100 annotated images per dataset. These results are supported by the Precision vs. Recall plot, where AUC is superior to 0.85 for all dataset.

Recall and precision per class are also presented in Table 4. Neutrophils are consistently classified with high precision and recall, whereas monocytes are often classified as lymphocytes and neutrophils. Recall is particularly low for basophils in the Munich 2018, 2021, and Rabin datasets. This result was expected, basophils being the rarest class.

**Table 4:** Precision (Pre) and Recall (Rec) per class after fine-tuning the model for every *target dataset*. Precision and recall are computed, respectively, to their class.

Dataset	Bas		Eos		Ery		Lym		Mon		Neu	
	Pre	Rec	Pre	Rec	Pre	Rec	Pre	Rec	Pre	Rec	Pre	Rec
Rabin	0.88	0.36	0.78	0.72			0.87	0.97	0.73	0.79	0.96	0.98
Munich 2021	0.64	0.13	0.75	0.80	0.78	0.93	0.76	0.88	0.91	0.28	0.89	0.96
Lisc	0.96	1.0	1.0	0.70			0.96	0.93	0.70	0.97	0.88	0.91
Jslh					1.00	1.00					1.00	1.00
Jin woo choi					1.0	0.87					0.72	1.0
Jiangxi tecom			0.58	1.0			0.80	0.60	0.88	0.85	0.96	0.97
Bccd			0.70	0.67			0.94	0.94	0.81	0.92	0.77	0.71
Tianjin	0.95	0.73	0.81	0.99			0.77	0.94	0.93	0.92	0.98	0.98

Each class is represented in at least five datasets, except for erythroblasts which are only in three datasets. For this class, recall and precision are higher than 0.8 over all three datasets, including Munich 2021 dataset which contains 27,395 erythroblasts from 700 patients. We thus conclude that generalization capacity of our workflow also applies to erythroblasts.

Interestingly, performances of the model are not the highest for the Munich 2021 dataset, even if one of the *source datasets* comes from the same laboratory. This can be explained by various factors: cells come from bone marrow in one dataset and from peripheral blood in the other, and the conditions of acquisition are significantly different (camera, magnification power, resolution, format of the image, date). This contributed to make the visual aspects of these two datasets drastically different.

### Ablation study

To better understand the contribution of each step of our approach, we carried out a series of complementary experiments. The comparison was drawn between the following experimental settings.

1) **Without source train**; we directly trained a new model on each dataset, without any pretraining on the *source* dataset.
2) **Without fine-tuning**; the model that was trained on the *source* dataset was directly tested on images from the *target* dataset.
3) **Head only fine-tuning**; only the head of the model was fine-tuned, the encoder was frozen.
4) **Mixed sources**; the model was fine-tuned with images from mixed sources, i.e., from both the *target* dataset and the *source* dataset (100 + 100 images from Barcelona + Tianjin).
5) **Fine-tuning without color transform**; the model was fine-tuned without applying color transform on target images.
6) **Proposed workflow**; the model was fine-tuned under the previously described experimental conditions (c.f. workflow presented in “Methods”).

The ablation study was conducted for each dataset, and the average performances are presented in Table 5. Detailed results can be found in Appendix C. We calculated *p*-values using a pairwise Wilcoxon test, demonstrating that the differences in accuracy observed between two experimental conditions were significant. The outcomes of this study confirm the superiority of our workflow over other experimental setups. Furthermore, it can be noticed that performance improvement comes principally from fine-tuning. The addition of color transform only slightly influences results; in general, fine-tuning is more important than color transform to improve classification on images from a new dataset, even if their coloration is different.

**Table 5:** Classification results under various experimental conditions. The ablation study was performed for every dataset and then averaged. Accuracy is defined as the overall accuracy; computed with the total number of rightly classified images. Precision and recall are the average of precision and recall over the six classes (i.e., macro-measures). *p*-values are computed between every pair of successive experimental conditions (Condition 1 with Condition 2, Condition 2 with Condition 3…etc.). *p*-values lower than 1e-5 are approximated as ∼0.

Experimental conditions	Accuracy	Precision	Recall	*p* value
1.Without source train	0.48	0.38	0.34	
2. Without fine-tuning	0.59	0.48	0.50	∼0
3. Head only fine-tuning	0.71	0.65	0.60	∼0
4. Mixed sources fine-tuning	0.74	0.62	0.58	0.024
5. Fine-tuning without color transform	0.84	0.83	0.76	∼0
6. Proposed workflow	0.89	0.85	0.84	∼0

Additionally, a study on the influence of the fine-tuning sample size on accuracy, precision, and recall is provided in Appendix D. This analysis was conducted on the four largest datasets, each containing more than 1000 images, where 100 images were used for fine-tuning. We observed that the number of samples had only a small effect on final accuracy. However, it positively impacted the precision and recall of rare classes, such as eosinophils and basophils, in the Rabin dataset. In conclusion, we selected a sample size of 100 images for this study. Increasing the number would have significantly prolonged the annotation process, whereas reducing it would have lowered the diversity of images in certain classes. For the smaller datasets, we had no choice but to use 1/10th of the dataset for fine-tuning while preserving enough images for evaluation.

### Relevance of color transformation

**Fig. 8:**
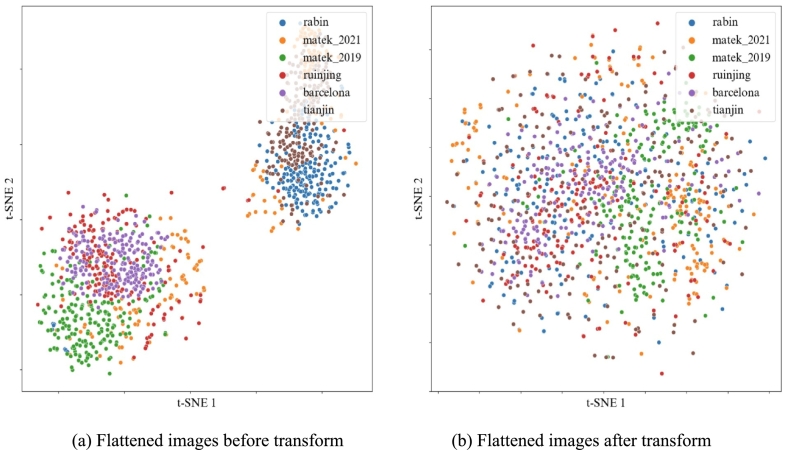
t-SNE representation of images from different datasets before and after transform. Each point is obtained by flattening the corresponding image and performing dimensionality reduction. For each dataset, only 200 images are represented.

Visual transformation aimed to reduce color disparities resulting from the variety of possible imaging conditions. This transform presented several advantages, including computational efficiency, alignment with color standards familiar to clinicians, and the prevention of image distortion. The representation of images from each dataset is shown in Fig. 8, which evidenced the impact of transform. Each point corresponded to a flattened image after t-SNE dimensionality reduction.^43^ After transform, the distributions of pixels in 2D overlapped better among different datasets.

**Fig. 9:**
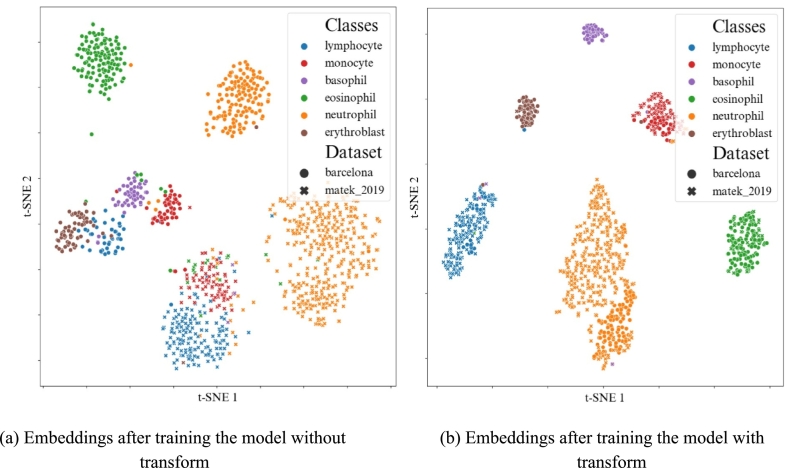
t-SNE of the embeddings output by the encoder of the model, after the model was trained with or without the color transform. Barcelona images are represented with dots, whereas Munich 2018 images are represented with crosses. The transform brings together images from different datasets when they belong to the same class.

In general, color transform was efficient at reducing domain shift, at a very low cost. In Fig. 9, the embeddings of the images from the *source dataset* were visualized following a t-SNE dimensionality reduction. The plots illustrated how the transform function could bring images from different datasets closer together when they belonged to the same class. Thus, the use of a transform yielded tighter clusters of classes irrespective of the image's source dataset. The workflow was a form of domain adaptation method.

### Relevance of fine-tuning

**Fig. 10:**
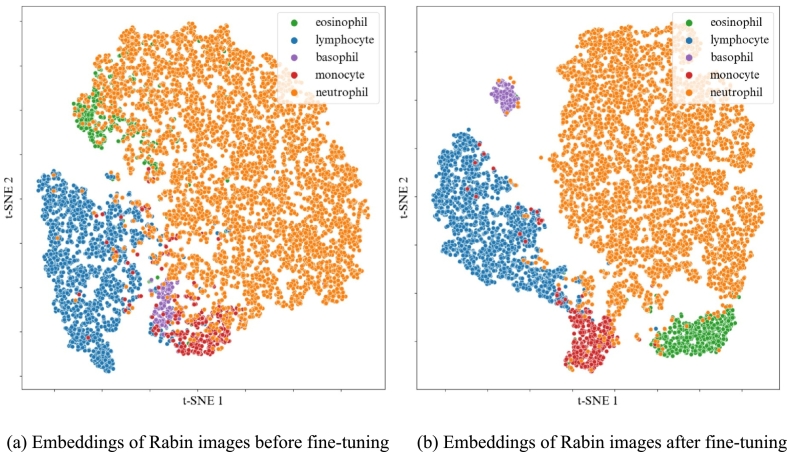
t-SNE of the embeddings output by the encoder of the model, before and after fine-tuning the model with 100 annotated images from the Rabin dataset.

The fine-tuning strategy leveraged classification performances on the *target* datasets, enabling satisfactory results even with a limited number of annotated images. In Fig. 10, the visualization of embeddings from Rabin images illustrated how fine-tuning could improve classification performances when applied to a *target* dataset. Notably, fine-tuning allowed a better recognition of basophils, monocytes and eosinophils. Given that color plays a decisive role in identifying these cell categories, we can suppose that fine-tuning leads to a better comprehension of the coloration in the newly encountered dataset. Additionally, it allowed the classification to be very fast on large datasets, with the fine-tuning process completed quickly, and requiring the labeling of only 100 images by experts.

### Wrong-prediction analysis

**Fig. 11:**
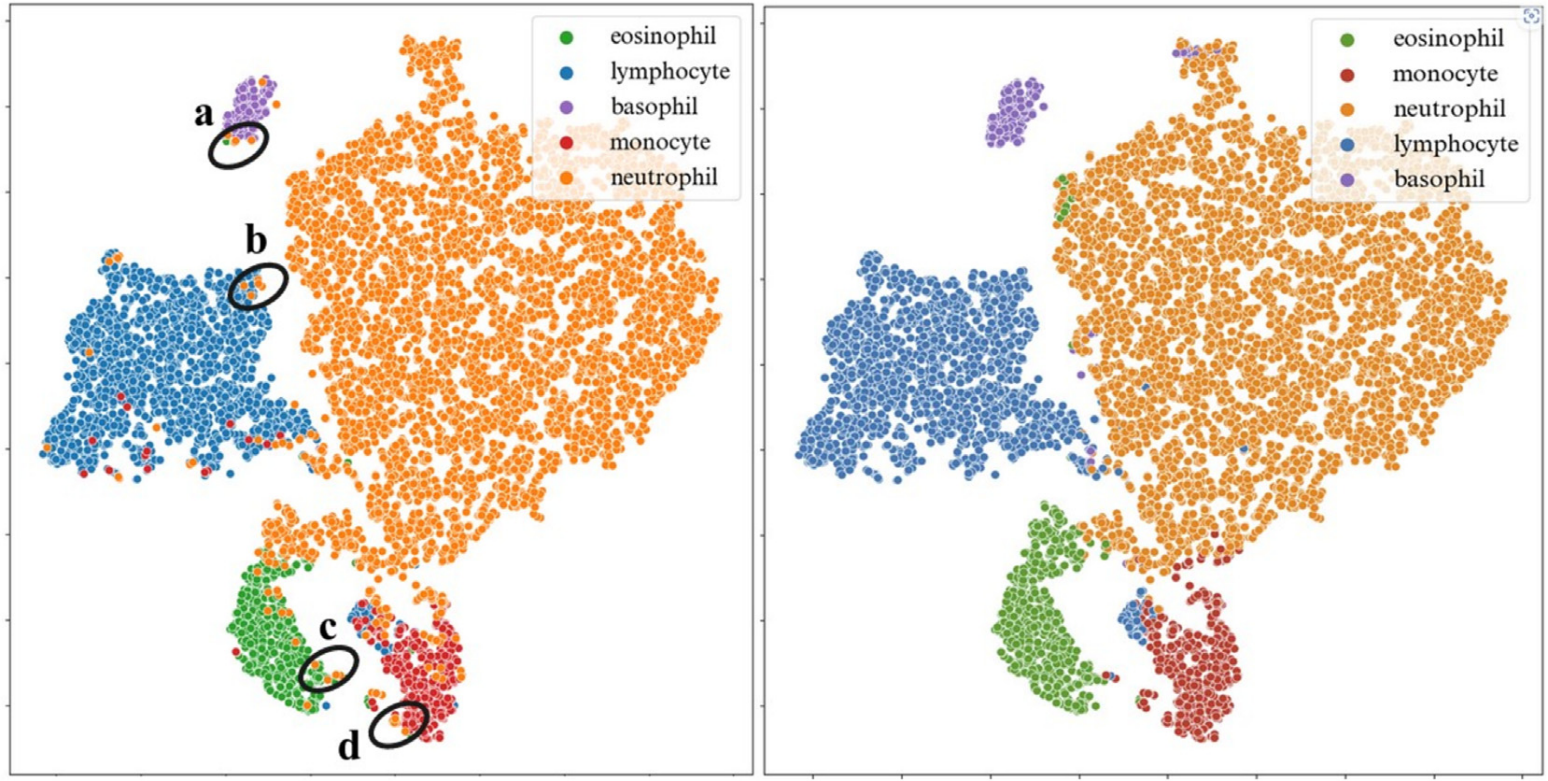
Embedding space projected in 2D with t-SNE. Colors on the left correspond to ground truth labels, and colors on the right are rightly predicted labels. We studied wrongly predicted neutrophils from each dark circle region.

**Fig. 12:**
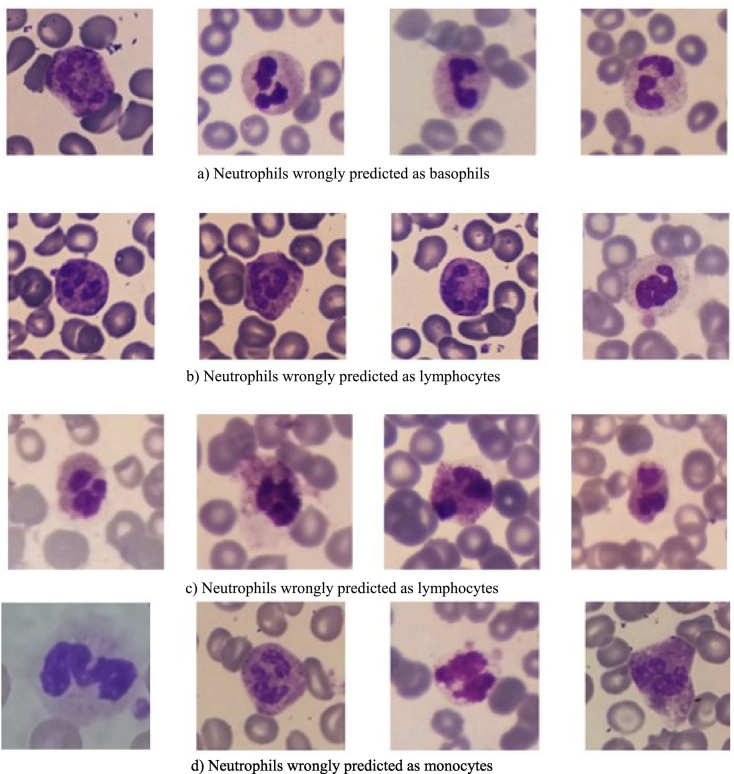
Analysis of images of neutrophils wrongly predicted in each category. Factors contributing to prediction errors in each category include image quality, granulation color, nucleus shape, and cytoplasm color.

To have a deeper understanding of these results, we conducted an analysis of wrong predictions on Rabin dataset as depicted in Fig. 11, Fig. 12. Our focus was directed towards wrongly classified neutrophils. We showed their corresponding representation within the embedding space using a black circle. Beside the possible impact of nucleus shape, we observe that neutrophils showing purple granulation are inaccurately categorized as basophils, whereas those with more orange granulations are wrongly identified as eosinophils. Neutrophils featuring an indistinct nucleus and blurred granulation are often misclassified as lymphocytes. Additionally, neutrophils presenting a blue coloration in their cytoplasm frequently lead to mispredictions of monocytes. It is worth noticing that t-SNE clusters are not exactly aligned with the predictions, and there are a few exceptions of images belonging to a cluster but being predicted in another category (e.g., eosinophils in neutrophil cluster in Fig. 11 right).

**Fig. 13:**
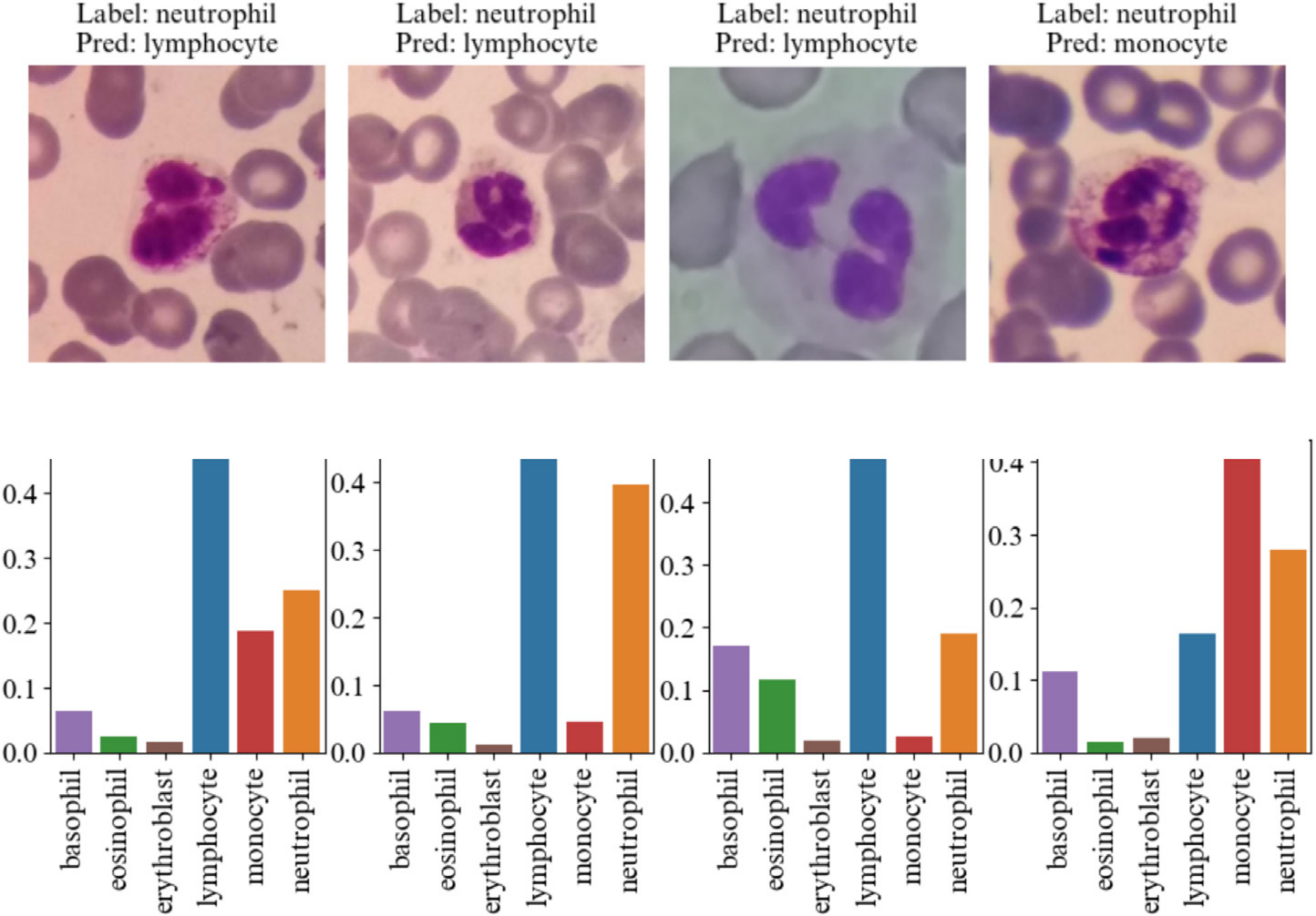
Output probability vector for wrongly predicted neutrophils by the model. Even if the highest probability is given to a wrong prediction label, the second highest probability corresponds neutrophil, which aligns with the true class of the cell.

To conclude this analysis, we examined the output probability vector for incorrectly predicted neutrophils (Fig. 13). In the majority of wrong predictions, the second highest probability corresponds neutrophil, which aligns with the true class of the cell.

### Limitations and perspectives

The results of this study exhibit some limitations. Firstly, whereas the results were promising in terms of generalizability, further research would be necessary to improve classification performances for the clinical implementation of this algorithm. This could be achieved by using active learning: images where the model hesitates between different classes as in Fig. 13 could be re-annotated by the clinician to improve performances. Introducing human-in-the-loop annotation could overcome this limitation.

Secondly, the diverse datasets presented highly imbalanced cell distributions. Whereas some target datasets were large and exhibited a wide range of cell types, others consisted of only two classes or contained only a very limited number of cells. We did not discard small or heavily unbalanced datasets based on a minimum number of images criterion, as it appeared that they were accepted and used by the expert community.^11^^,^^16^^,^^20^^,^^26^ However, the representativeness of the results for these datasets should be studied to a greater extent. Although this diversity shows that our approach leads to a good capacity for generalization, some representations do not align with the diversity observed in real blood cell populations. In addition, further research should confirm these results with a broader system of classes, including earlier stages of white blood cell maturation that are commonly observed in bone marrow samples.

Lastly, the study of wrong predictions by the algorithm has underscored the importance of accurate annotation and high-quality images. The algorithm faced particular challenges when cells exhibited granulations that low resolution failed to display, or when there was there was an ambiguity about the cell's ground truth label (see Appendix E).

Nonetheless, this study introduced an easy-to-implement workflow to obtain robust classification performances. This was the first study to explore the generalizability capacity of models at low computational cost, and with so many datasets. The workflow was elaborated by taking into consideration the diversity of coloration images could take. Furthermore, it was specifically designed to be fast in computations, and to cut down on the necessary annotation time. Hence, this article provides an approach that can be used on images with various visual aspect and was applied to a local dataset with good performances.

## Conclusion

In this study, we proposed a workflow to enable a classification EfficientNet network to accurately label cells from 10 different sources with low computational cost. The key aspect of our approach lies in combining a color transformation to standardize the visual style of images with a fine-tuning technique. Fine-tuning significantly improved classification performance, resulting in an overall accuracy exceeding 80% for all datasets. Furthermore, the benefits of fine-tuning and color transformation were visually confirmed by plotting the embeddings of the images generated by the model, highlighting the model's improved ability to cluster images by cell classes.

Further research should be conducted, firstly to enhance classification accuracy for specific datasets and secondly to create larger and more diverse datasets of cell images.

## Funding source

This research did not receive any specific grant from funding agencies in the public, commercial, or not-for-profit sectors.

## Declaration of competing interest

The authors declare that they have no known competing financial interests or personal relationships that could have appeared to influence the work reported in this article.

## Figures and Tables

**Table A.1:** Dataset detailed description.

Name	Centre	Nb	Classes	Imaging system and format	Magnification	PBS or BMS	Healthy or pathological
Barcelona^31^	Core Laboratory the Hospital Clinic, Barcelona	17,092	NEU, EOS, BAS, LYT, MON, PMO, MYB, EBO, PLA, THO	CellaVision DM96 (.jpg)	0.1 μm per pixel (360 × 363 pixels)	PBS	Healthy
Cella Vision Blog^33^		100	BAS, EOS, LYT, NEU, MON	CellaVision (.bmp)	(300 × 300 pixels)		
Jiangxi Tecom^33^	Jiangxi Tecom Science Corporation	300	BAS, EOS, LYT, NEU, MON	Motic Moticam Pro 252A optical microscope camera (.bmp)	(120 × 120)		
Jin Woo Choi^34^	Seoul National University Hospital.	2174	NGB, NGS, ERB, MMZ, MYO, MYB, ERO, ERP, PMO, PBL	(.jpg)	x1000 magnification, (144 × 144 pixels)	BMS	
BCCD^35^	Roboflow	3500	EOS LYT, MON, NEU	(.jpeg)	(320 × 240 pixels)	PBS	
LISC^36^	Hematology-Oncology and BMT Research Center of Imam Khomeini hospital in Tehran	257	BAS, EOS, LYT, NEU, MON	Light microscope (Microscope-Axioskope 40) and digital camera Sony Model No. SSCDC50AP(.png)	x100 (250 × 250 pixels)	PBS	Healthy
Munich 2021^4^	Helmholtz Zentrum München–German Research Center for Environmental Health,	171,373	NGB, NGS, LYT, MON, EOS, BAS, MMZ, MYB, PMO, BLA, PLM, KSC, OTH, ART, NIF, PEB, EBO, HAC, ABE, LYI, FGC	CCD camera mounted on a brightfield microscope (Zeiss Axio Imager Z2) (.jpg)	x40 (250 × 250 pixels)	BMS	Pathological
Munich 2019^32^	Helmholtz Zentrum München–German Research Center for Environmental Health,	18,365	BAS, EBO, EOS, KSC, LYT, LYA, MMZ, MYO, MON, NGB, NGS, PMB	M8 digital microscope Scanner (Precipoint GmbH, Freising) (.tiff)	x100 (400 × 400 pixels)	PBS	Pathological
Raabin^37^	Razi Hospital in Rasht, Gholhak Laboratory, Shahr-e-Qods Laboratory, and Takht-e Tavous Laboratory in Tehran,	16,633	EOS, LYT, MON, NEU	Smartphones mounted to occular lenses of microscopes (Olympus CX18 and Zeiss) (.jpg)	x100 (575 × 575 pixels)	PBS	Both
Ruinjing^44^	Ruijing Hospital, Shanghai Jiaotong University School of Medicine	1647	BLA, LYT, LYA	Cella Vision (.jpg)	x100 (360 × 363)	PBS	Pathological
JSLH^38^	Japan Society of Diagnostics Hemtaology	148	NGB, ERB, MMZ, MYO, ERO, ERP, PEB, PMO, NGS	(.png)	(85 × 74 pixels)		
Tianjin^39^	Tianjin Medical University Affiliated Medical Center	6228	BAS, EOS, LYT, NEU, MON	Nikon DS-Ri2 Color Camera (.png)	x100 (250 × 250 pixels)	PBS	

**Fig. B.1:**
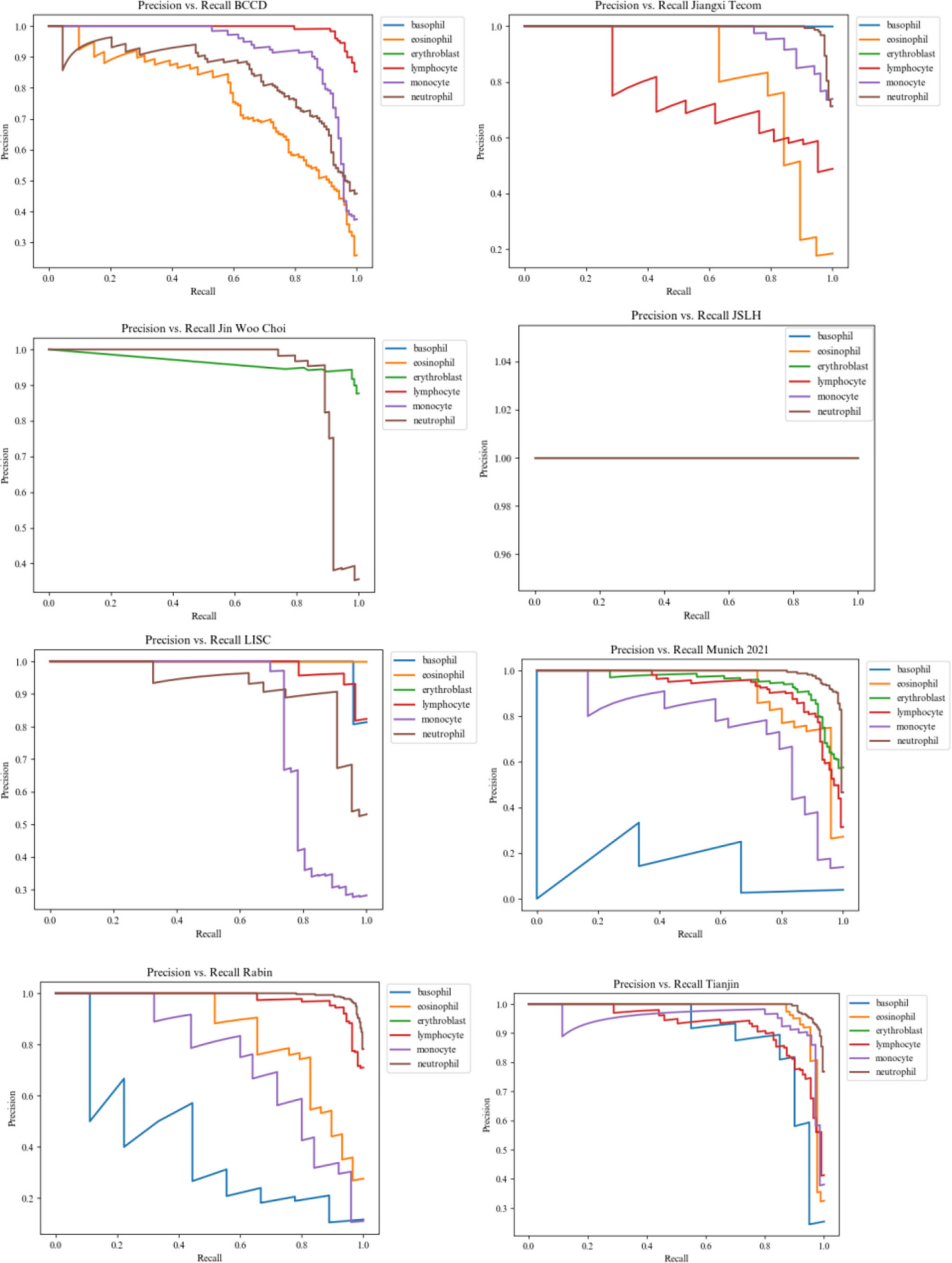
Precision–recall curve per class for each dataset.

**Table C.1:** Results under various experimental conditions for each dataset.

Experimental conditions	Dataset	Accuracy	Precision	Recall
1) no_source_train	Munich 2021	0.31	0.34	0.29
2) no fine_tune	Munich 2021	0.75	0.69	0.63
3) head_only	Munich 2021	0.77	0.74	0.62
4) mixed_train	Munich 2021	0.78	0.74	0.63
5) no_transform	Munich 2021	0.83	0.78	0.65
6) our_workflow	Munich 2021	0.82	0.79	0.66

1) no_source_train	Rabin	0.49	0.48	0.34
2) no fine_tune	Rabin	0.65	0.47	0.46
3) head_only	Rabin	0.82	0.85	0.67
4) mixed_train	Rabin	0.80	0.70	0.55
5) no_transform	Rabin	0.92	0.76	0.68
6) our_workflow	Rabin	0.92	0.85	0.76

1) no_source_train	LISC	0.56	0.46	0.50
2) no fine_tune	LISC	0.46	0.38	0.45
3) head_only	LISC	0.67	0.68	0.67
4) mixed_train	LISC	0.71	0.71	0.70
5) no_transform	LISC	0.93	0.93	0.93
6) our_workflow	LISC	0.89	0.90	0.90

1) no_source_train	JSLH	0.45	0.18	0.25
2) no fine_tune	JSLH	1.00	1.00	1.00
3) head_only	JSLH	1.00	1.00	1.00
4) mixed_train	JSLH	1.00	1.00	1.00
5) no_transform	JSLH	1.00	1.00	1.00
6) our_workflow	JSLH	1.00	1.00	1.00

1) no_source_train	Jin Woo Choi	0.47	0.16	0.22
2) no fine_tune	Jin Woo Choi	0.57	0.18	0.31
3) head_only	Jin Woo Choi	0.81	0.55	0.54
4) mixed_train	Jin Woo Choi	0.78	0.32	0.33
5) no_transform	Jin Woo Choi	0.88	0.89	0.86
6) our_workflow	Jin Woo Choi	0.90	0.86	0.94

1) no_source_train	Jiangxi Tecom	0.69	0.64	0.47
2) no fine_tune	Jiangxi Tecom	0.16	0.20	0.21
3) head_only	Jiangxi Tecom	0.85	0.51	0.47
4) mixed_train	Jiangxi Tecom	0.54	0.34	0.31
5) no_transform	Jiangxi Tecom	0.77	0.71	0.52
6) our_workflow	Jiangxi Tecom	0.90	0.85	0.88

1) no_source_train	BCCD	0.53	0.35	0.36
2) no fine_tune	BCCD	0.25	0.17	0.17
3) head_only	BCCD	0.41	0.40	0.40
4) mixed_train	BCCD	0.41	0.40	0.41
5) no_transform	BCCD	0.84	0.83	0.85
6) our_workflow	BCCD	0.81	0.81	0.81

1) no_source_train	Tianjin	0.33	0.41	0.27
2) no fine_tune	Tianjin	0.85	0.69	0.77
3) head_only	Tianjin	0.82	0.86	0.77
4) mixed_train	Tianjin	0.91	0.76	0.71
5) no_transform	Tianjin	0.56	0.73	0.62
6) our_workflow	Tianjin	0.91	0.74	0.76

**Fig. D.1:**
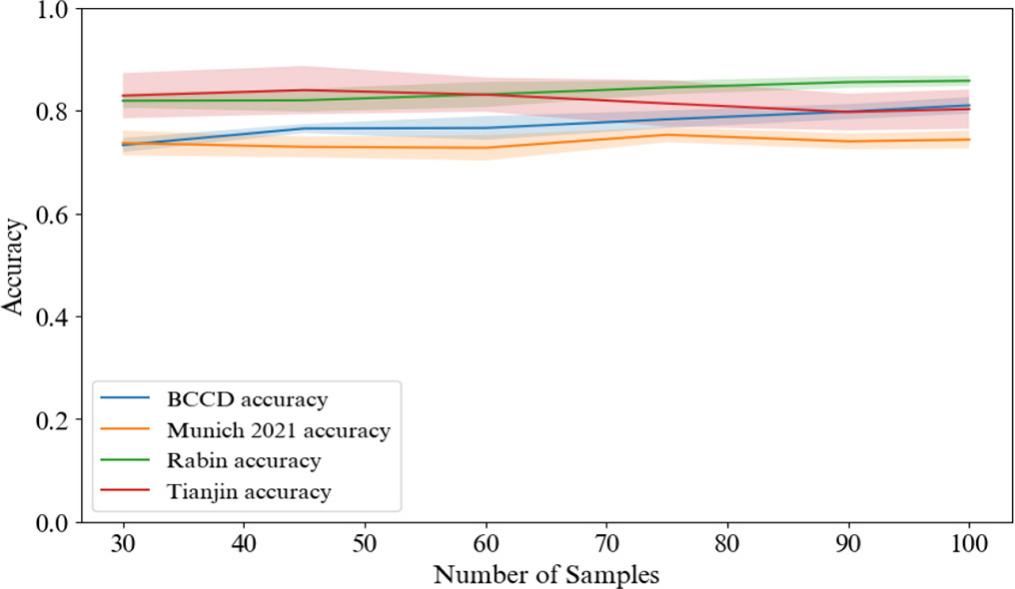
Evolution of the overall accuracy with the number of samples used for fine-tuning. For each sample size *n*ϵ [30, 45, 60, 75, 90, 100], we randomly picked n samples and reproduced the experiment 10 times. The curve corresponds to the mean accuracy, and the envelopes to the standard deviation of the results for each sample size *n* over the 10 experiments.

**Fig. D.2:**
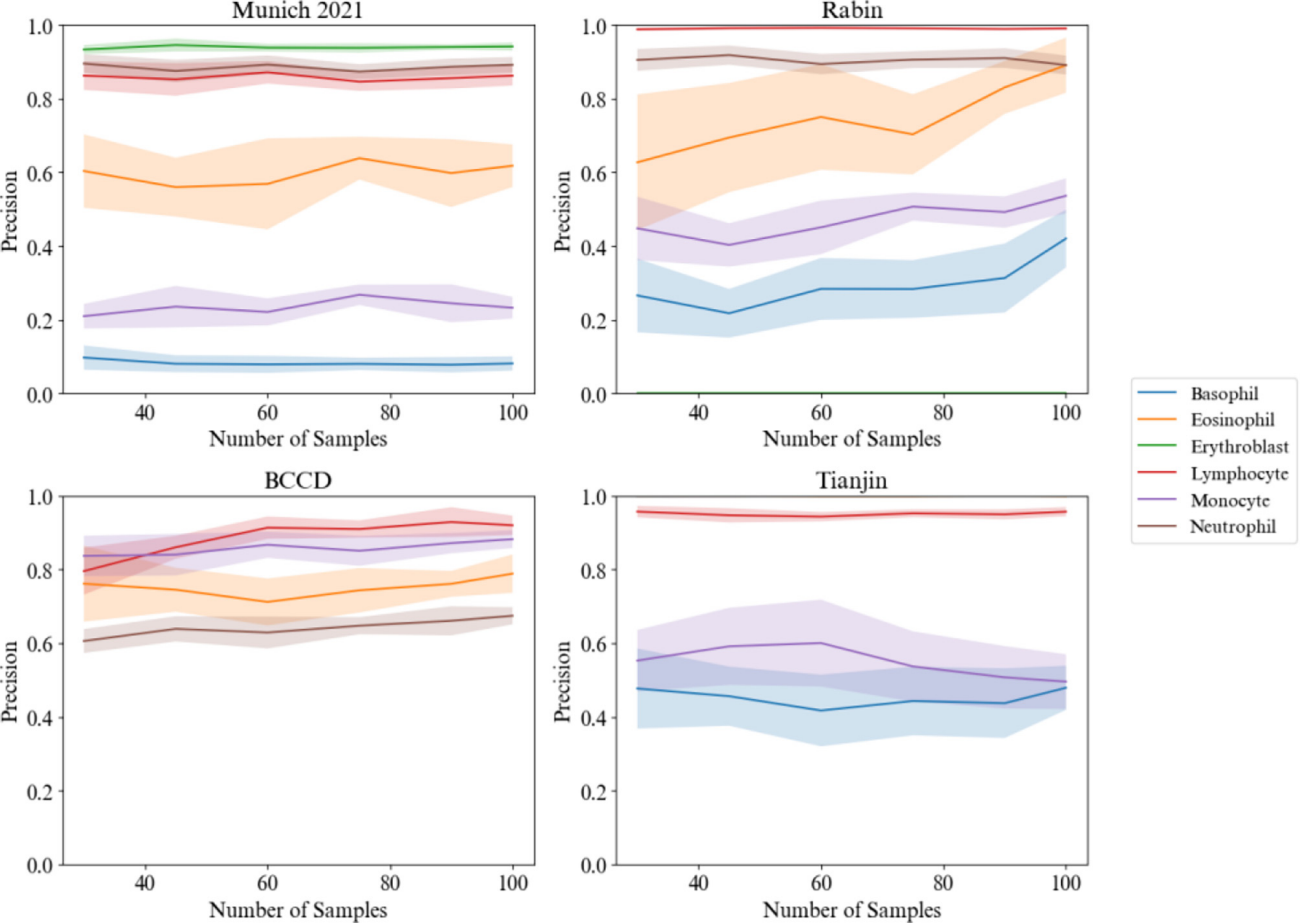
Evolution of the precision per class with the number of samples used for fine-tuning. Each experiment has been performed 10 time, the envelopes correspond to the standard deviation of the results.

**Fig. D.3:**
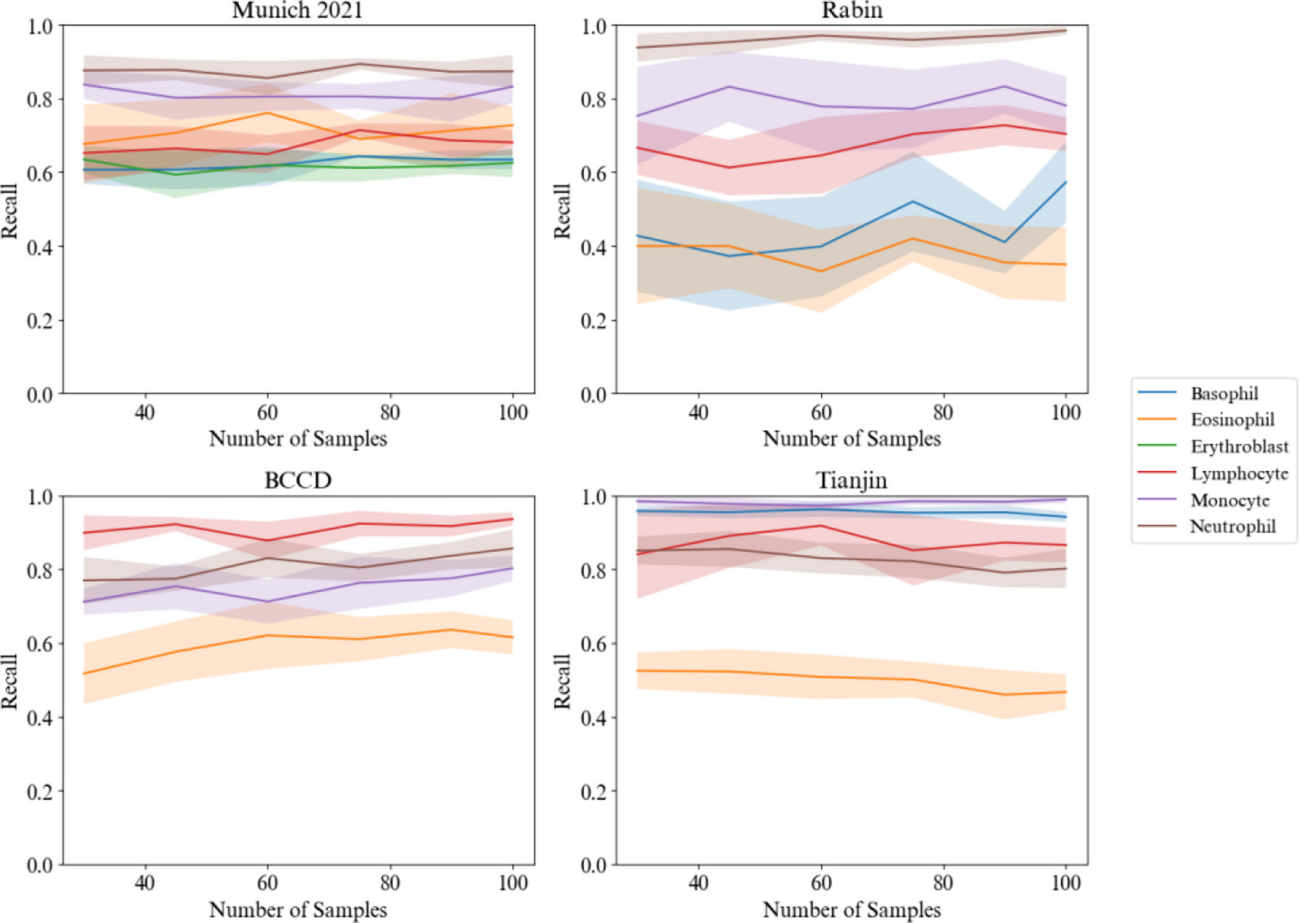
Evolution of the recall per class with the number of samples used for fine-tuning. Each experiment has been performed 10 time, the envelopes correspond to the standard deviation of the results.

**Fig. E.1:**
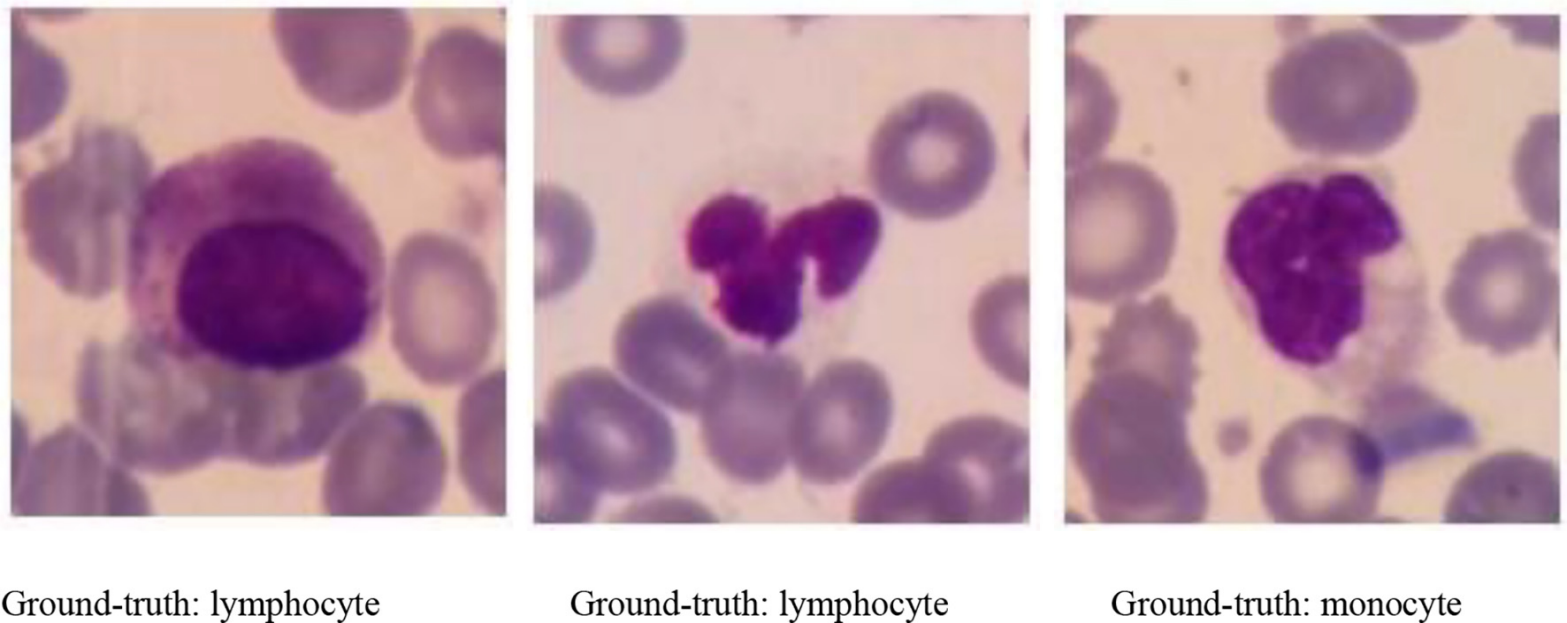
Examples of images from Rabin dataset with ambiguous ground-truth. According to the dataset ground-truth, these cells are lymphocytes and monocytes, but they present morphological properties of granulocytes (granulations, segmented nucleus).

## Glossary

Abbreviation: Meaning
ABE: Abnormal eosinophil
ART: Artifact
BAS: Basophil
BLA: Blast
BMS: Bone marrow smear
EBO: Erythroblast
EOS: Eosinophil
ERA: Acidophilic erythroblast
ERB: Basophilic erythroblast
ERO: Orthochromatic erythroblast
ERP: Polychromatic erythroblast
FGC: Faggott cell
GAN: Generative adversarial network
HAC: Hairy cell
KSC: Smudge cell
LYA: Lymphocyte (atypical)
LYA: Lymphoma
LYI: Immature lymphocyte
LYT: Lymphocyte
MGG: May-Grünwald Giemsa
MMZ: Metamyelocyte
MON: Monocyte
MYB: Myelocyte
MYO: Myeloblast
NEU: Neutrophil
NGB: Band neutrophil
NGS: Segmented neutrophil
NIF: Not identifiable
OTH: Other cell
PBL: Pronormoblast
PBS: Peripheral blood smear
PEB: Proerythroblast
PLA: Platelet
PLM: Plasma cell
PMB: Promyelocyte (bilobed)
PMO: Promyelocyte
THO: Thrombocyte
WBC: White blood cell
